# Additional Feeding Reveals Differences in Immune Recognition and Growth of *Plasmodium* Parasites in the Mosquito Host

**DOI:** 10.1128/mSphere.00136-21

**Published:** 2021-03-31

**Authors:** Hyeogsun Kwon, Maria L. Simões, Rebekah A. Reynolds, George Dimopoulos, Ryan C. Smith

**Affiliations:** a Department of Entomology, Iowa State University, Ames, Iowa, USA; b Department of Molecular Microbiology and Immunology, Johns Hopkins Bloomberg School of Public Health, Baltimore, Maryland, USA; University of Copenhagen

**Keywords:** blood feeding, host-pathogen interactions, immune evasion, innate immunity, malaria, mosquito

## Abstract

Mosquitoes may feed multiple times during their life span in addition to those times needed to acquire and transmit malaria. To determine the impact of subsequent blood feeding on parasite development in Anopheles gambiae, we examined Plasmodium parasite infection with or without an additional noninfected blood meal. We found that an additional blood meal significantly reduced Plasmodium berghei immature oocyst numbers, yet had no effect on the human parasite Plasmodium falciparum. These observations were reproduced when mosquitoes were fed an artificial protein meal, suggesting that parasite losses are independent of blood ingestion. We found that feeding with either a blood or protein meal compromises midgut basal lamina integrity as a result of the physical distention of the midgut, enabling the recognition and lysis of immature P. berghei oocysts by mosquito complement. Moreover, we demonstrate that additional feeding promotes P. falciparum oocyst growth, suggesting that human malaria parasites exploit host resources provided with blood feeding to accelerate their growth. This is in contrast to experiments with P. berghei, where the size of surviving oocysts is independent of an additional blood meal. Together, these data demonstrate distinct differences in *Plasmodium* species in evading immune detection and utilizing host resources at the oocyst stage, representing an additional, yet unexplored component of vectorial capacity that has important implications for the transmission of malaria.

**IMPORTANCE** Mosquitoes must blood feed multiple times to acquire and transmit malaria. However, the impact of an additional mosquito blood meal following malaria parasite infection has not been closely examined. Here, we demonstrate that additional feeding affects mosquito vector competence; namely, additional feeding significantly limits Plasmodium berghei infection, yet has no effect on infection of the human parasite P. falciparum. Our experiments support that these killing responses are mediated by the physical distension of the midgut and by temporary damage to the midgut basal lamina that exposes immature P. berghei oocysts to mosquito complement, while human malaria parasites are able to evade these killing mechanisms. In addition, we provide evidence that additional feeding promotes P. falciparum oocyst growth. This is in contrast to P. berghei, where oocyst size is independent of an additional blood meal. This suggests that human malaria parasites are able to exploit host resources provided by an additional feeding to accelerate their growth. In summary, our data highlight distinct differences in malaria parasite species in evading immune recognition and adapting to mosquito blood feeding. These observations have important, yet previously unexplored, implications for the impact of multiple blood meals on the transmission of malaria.

## INTRODUCTION

Blood feeding is an inherent behavior of all hematophagous arthropods that provides nutritional resources for development or reproduction while enabling the acquisition and transmission of a pathogen from one host to the next. This includes a number of arthropod-borne diseases that influence human health, most notably malaria, which causes more than 200 million infections and 400,000 deaths every year (1). Caused by Plasmodium parasites, malaria transmission requires the bite of an Anopheles mosquito, and therefore understanding the factors that influence vectorial capacity are integral to efforts to reduce malaria transmission.

Following the ingestion of an infectious blood meal, malaria parasites undergo substantial development in the mosquito host as they transition from gametes to a fertilized zygote, a motile ookinete, an oocyst, and a sporozoite capable of transmission to a new host (2). During this approximately 2-week period of development (referred to as the extrinsic incubation period [EIP]), significant bottlenecks reduce parasite numbers at each of these respective *Plasmodium* stages (2). These losses are mediated in part by the mosquito innate immune system that targets the *Plasmodium* ookinete or oocyst through distinct immune mechanisms (3–6). However, for those parasites that are able to escape immune recognition, the influence of changes to mosquito physiology for the remainder of the parasite life cycle remains unknown. This includes nutritional stress (i.e., starvation and dehydration) and the potential for multiple blood meals before *Plasmodium* sporozoites reach the salivary glands of the mosquito, which could significantly impact the EIP and the likelihood of transmission (7).

With the ability to complete a gonotrophic cycle approximately every 3 days, mosquitoes can feed multiple times during their life span. Therefore, the consequences of additional feeding behaviors following an initial infection are crucial to our understanding of malaria transmission, yet have only been addressed in limited studies. It has been suggested that *Plasmodium*-infected mosquitoes were more likely to seek an additional blood meal (8), while others have demonstrated that additional feeding increased sporozoite infection of the mosquito salivary glands (9–11). However, the impacts of a blood meal on developing *Plasmodium* parasites have not been fully explored.

Here, we examine the influence of an additional blood meal on survival and development of *Plasmodium* oocysts, the malaria parasite stage most directly impacted by subsequent blood feeding after an initial infection. Performing experiments with both rodent and human malaria parasites, we see distinct differences in parasite survival and growth in response to additional feeding. Our results suggest that Plasmodium falciparum oocysts have evolved mechanisms to evade immune detection and to capture host resources to facilitate their growth, arguing that an additional blood feeding increases the likelihood of malaria transmission. Together, these findings provide novel insight into the host-parasite interactions that determine vectorial capacity and define important new implications for the role of mosquito feeding behavior in the efficacy of malaria transmission.

## RESULTS

### Additional feeding differentially affects *Plasmodium* survival.

**FIG 1:**
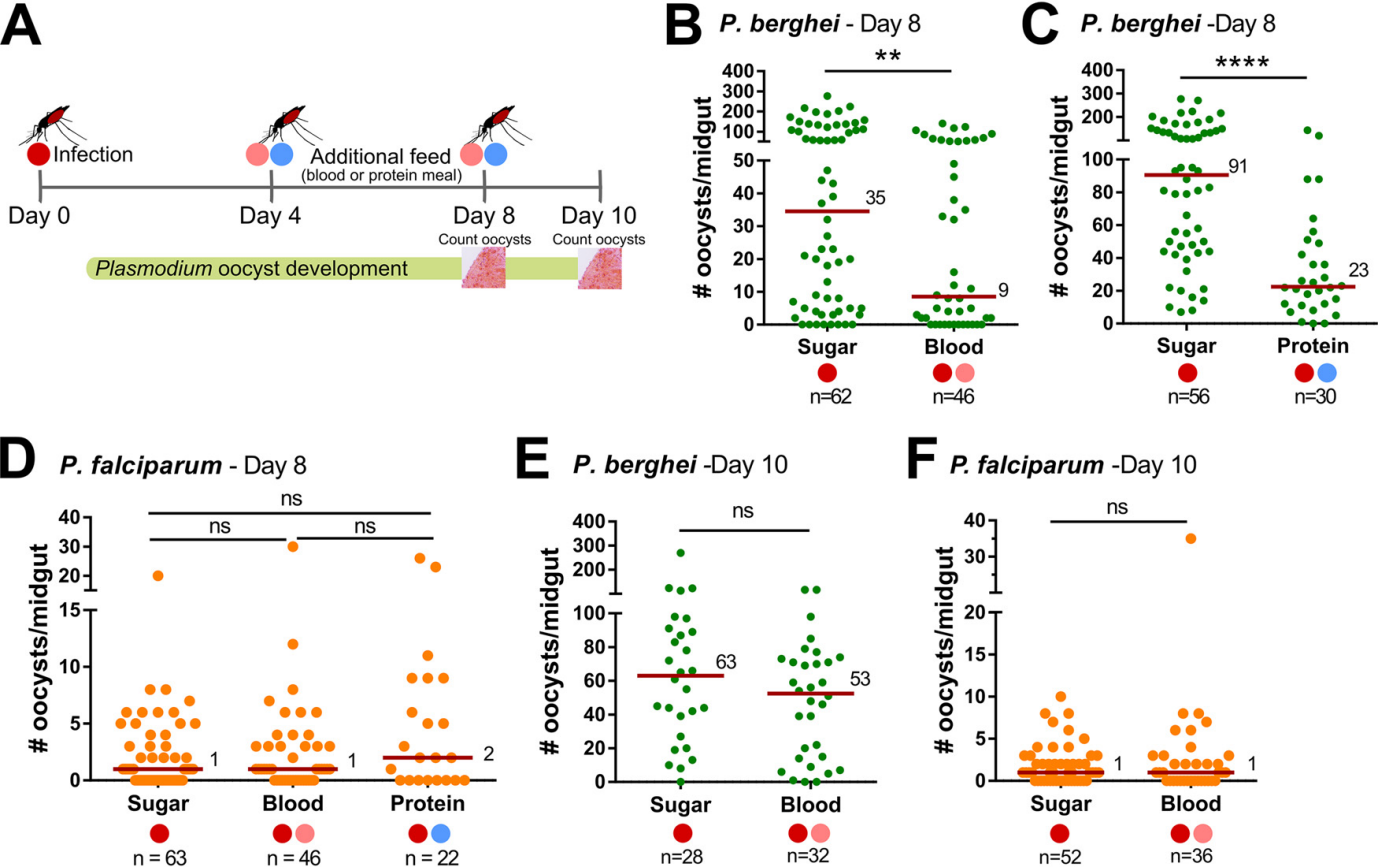
Additional feeding differentially impacts rodent and human malaria parasite survival. Experimental overview of additional feeding experiments in A. gambiae (A). Following an initial infection (day 0) with Plasmodium berghei or Plasmodium falciparum (dark red circle), blood fed mosquitoes were maintained on sugar or were challenged with an additional blood (pink circle) or protein (blue circle) meal at either 4 or 8 days postinfection. The effects of each experimental condition on oocyst numbers were then evaluated at 8 or 10 days postinfection, respectively. The influence of an additional feeding on P. berghei oocyst numbers was examined on day 8 for mosquitoes receiving an additional blood (B) or protein meal (C) 4 days postinfection. Similar experiments were also performed with P. falciparum, where oocyst numbers were evaluated on day 8 in mosquitoes that received an additional blood or protein meal 4 days postinfection (D). Potential temporal effects of the timing of feeding on oocyst survival were also examined, where mosquitoes infected with P. berghei or P. falciparum were maintained on sugar or received an additional uninfected blood meal 8 days postinfection (E and F). Oocyst numbers were evaluated at 10 days postinfection for P. berghei (E) and P. falciparum (F). For all experiments, each dot represents the number of parasites on an individual midgut, with the median value denoted by a horizontal red line. Mosquito infection data were pooled from three or more independent experiments (B to D) or from two independent experiments (E and F). Statistical analysis was performed using nonparametric tests for individual comparisons (Mann-Whitney U) or multiple comparisons (Kruskal-Wallis with a Dunn’s multiple-comparison test) using GraphPad Prism 7 software. Asterisks denote significance (**, *P < *0.01; ****, *P < *0.0001). *n*, number of mosquitoes examined per group; ns, not significant.

To examine the effects of an additional blood meal on malaria parasite infection, our methodology was to first infect Anopheles gambiae (Keele) mosquitoes with either Plasmodium berghei or Plasmodium falciparum, then to maintain one cohort on sugar while the second received an additional feed (naive blood or a protein meal) at either 4 or 8 days postinfection (Fig. 1A; see also Fig. S1 in the supplemental material). Using this methodology, we could evaluate the effects of an additional feeding on survival and development of *Plasmodium* oocysts, the parasite life stage most influenced by changes in mosquito physiology due to its approximately 2-week period of development. In experiments with mosquitoes with an established P. berghei infection, mosquitoes receiving a second blood meal 4 days postinfection had significantly reduced oocyst numbers (*P < *0.01) compared to those of control mosquitoes maintained only on sugar after the initial infection (Fig. 1B; see also Table S1 in the supplemental material).

10.1128/mSphere.00136-21.1 FIG S1 Experimental overview of additional feeding studies. Experimental overview of feeding experiments where Anopheles gambiae mosquitoes were initially challenged with Plasmodium berghei (A) or Plasmodium falciparum (B). They were then either maintained on sugar or received an additional uninfected blood or protein meal 4 days postinfection. For both panels A and B, oocyst numbers were examined at 8 days postinfection. Similar experiments were performed for both P. berghei or P. falciparum mosquitoes (C) in which an additional blood meal was provided at 8 days postinfection. Oocyst numbers were evaluated at 10 days postinfection. Download FIG S1, TIF file, 2.9 MB. Copyright © 2021 Kwon et al. 2021 Kwon et al. https://creativecommons.org/licenses/by/4.0/ This content is distributed under the terms of the Creative Commons Attribution 4.0 International license.

10.1128/mSphere.00136-21.3 TABLE S1 Raw data for additional blood- and protein-feeding experiments. Download Table S1, XLSX file, 0.02 MB. Copyright © 2021 Kwon et al. 2021 Kwon et al. https://creativecommons.org/licenses/by/4.0/ This content is distributed under the terms of the Creative Commons Attribution 4.0 International license.

Additional experiments were performed using simplified nutritional sources that would similarly promote midgut distention comparable to blood feeding. Previous studies have demonstrated that this can be achieved through the use of a low-melt agarose diet (150 mM NaCl, 20 mM NaHCO3, 20 mM ATP, and 0.2% low-melt-temperature agarose) (12) or by feeding an artificial protein diet (2% bovine serum albumin [BSA] and 2 mM ATP in 1× phosphate-buffered saline [PBS]) (13). However, experiments providing an additional feeding with the low-melt agarose diet (similar to Fig. 1) resulted in 100% mosquito mortality (Fig. S2), likely due to the low incubation temperatures (19°C) for P. berghei, which are below the melting point of the low-melt agarose (∼25°C). Alternatively, additional feeding experiments were successfully performed using the artificial protein diet and a similar methodology to that of the blood-feeding experiments, in which one cohort was maintained on sugar after the initial P. berghei infection, while a second cohort was given a protein meal 4 days postinfection (Fig. 1A and Fig. S1). Following the additional protein feeding, oocyst numbers were similarly significantly reduced (*P < *0.0001; Fig. 1C and Table S1). Although not directly compared, the effects of an additional noninfectious blood or protein meal result in a comparable reduction in P. berghei oocyst numbers, suggesting that there is a conserved mechanism between experimental conditions that promotes parasite killing.

10.1128/mSphere.00136-21.2 FIG S2 Low-melt agarose causes mortality in P. berghei-infected mosquitoes. P. berghei-infected mosquitoes were challenged with an additional meal of low-melt agarose at various concentrations (0.05, 0.1, and 0.2%) 4 days postinfection. After feeding, mosquitoes were maintained at 19°C, the standard temperature for P. berghei infection. Mosquito mortality was assessed at 24 h for each concentration of low-melt agarose. Each condition resulted in complete mortality of all of the mosquitoes that fed on the low-melt agarose. *n*, number of mosquitoes examined per treatment. Download FIG S2, TIF file, 0.08 MB. Copyright © 2021 Kwon et al. 2021 Kwon et al. https://creativecommons.org/licenses/by/4.0/ This content is distributed under the terms of the Creative Commons Attribution 4.0 International license.

Using a similar methodology, we also examined the influence of an additional blood or protein meal on infection of the human malaria parasite, Plasmodium falciparum (Fig. 1A and Fig. S1). However, P. falciparum-infected mosquitoes receiving an additional blood or protein meal did not influence P. falciparum oocyst numbers (Fig. 1D and Table S1), suggesting that there are differences in the recognition and killing of these two *Plasmodium* species in the mosquito host following an additional feeding.

To determine if there is a temporal effect on the influence of an additional feeding, mosquitoes infected with either P. berghei or P. falciparum received an additional blood meal 8 days postinfection (Fig. 1A and Fig. S1), a time in which developing oocysts have reached maturity and have initiated sporogony (14). At this stage of oocyst development, an additional blood meal does not influence either P. berghei (Fig. 1E and Table S1) or P. falciparum (Fig. 1F) oocyst numbers, suggesting that there is a temporal component associated with the additional feeding responses that influence P. berghei survival.

### An additional blood or protein feeding promotes similar changes to the microbiota and host physiology.

**FIG 2:**
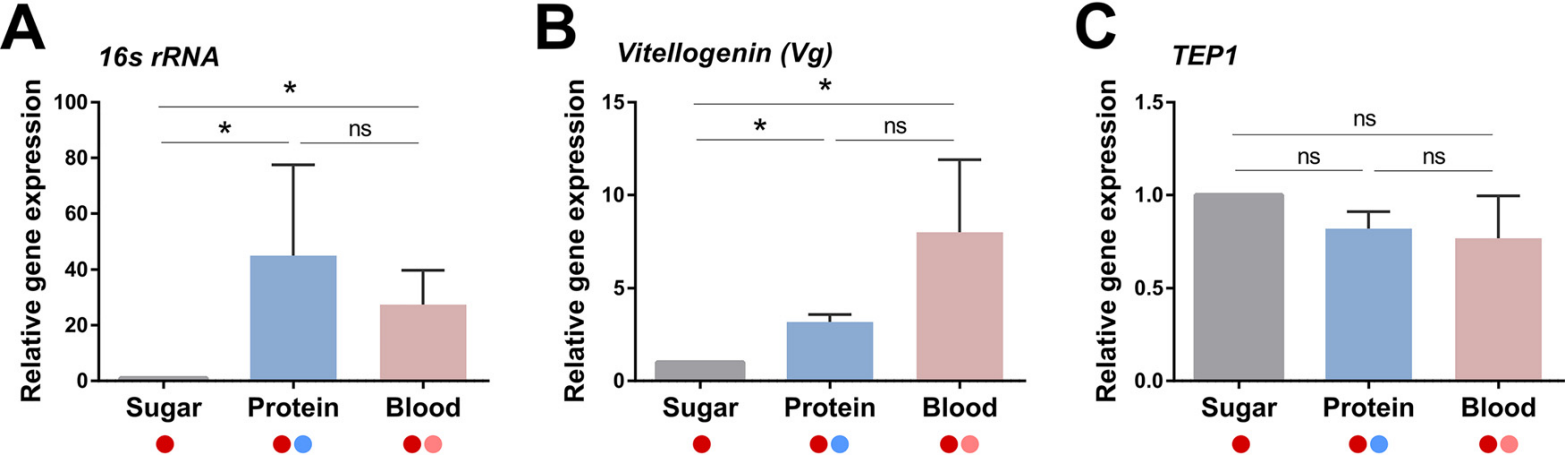
Additional protein or blood feeding similarly influence mosquito physiology. To examine the physiological impacts of additional feeding, P. berghei-infected mosquitoes (dark red circle) were challenged 4 days postinfection with an additional protein (blue circle) or blood meal (pink circle). Approximately 24 h after an additional feeding, samples were collected to examine the effects on the microbiome using bacterial 16S rRNA expression (A), vitellogenesis using vitellogenin (Vg) expression (B), and *TEP1* expression as a proxy for the immune system (C). Gene expression was examined by reverse transcription-quantitative PCR (qRT-PCR) using either dissected midgut samples (A) or whole-mosquito samples (B and C) using three or more independent biological replicates. Statistical analysis was performed using Mann-Whitney analysis with GraphPad Prism 7 software. Asterisks denote significance (*, *P < *0.05). ns, not significant.

Previous studies have demonstrated that artificial protein-based meals can stimulate physiological responses such as the proliferation of the midgut microbiota and vitellogenesis similar to blood feeding in the mosquitoes Aedes aegypti (15–17) and Anopheles coluzzii (18). To determine how additional feeding impacts the microbiota and host physiology of A. gambiae, mosquitoes were challenged with an initial infection with P. berghei and then given an additional protein or blood meal 4 days postinfection (Fig. 1). When we evaluated these samples by reverse transcription-quantitative PCR (qRT-PCR), both an artificial protein diet or blood meal promote proliferation of the midgut microbiota (Fig. 2A) and stimulate vitellogenin (Vg) expression (Fig. 2B), similarly to previous studies (15–18). This in contrast to *TEP1*, which does not display differences in expression between feeding conditions (Fig. 2C), suggesting that additional feeding does not enhance the mosquito complement response. Together, these data support additional feeding with blood or a minimal protein diet producing similar physiological responses in the mosquito host.

### Blood and protein feeding degrade the midgut basal lamina.

**FIG 3:**
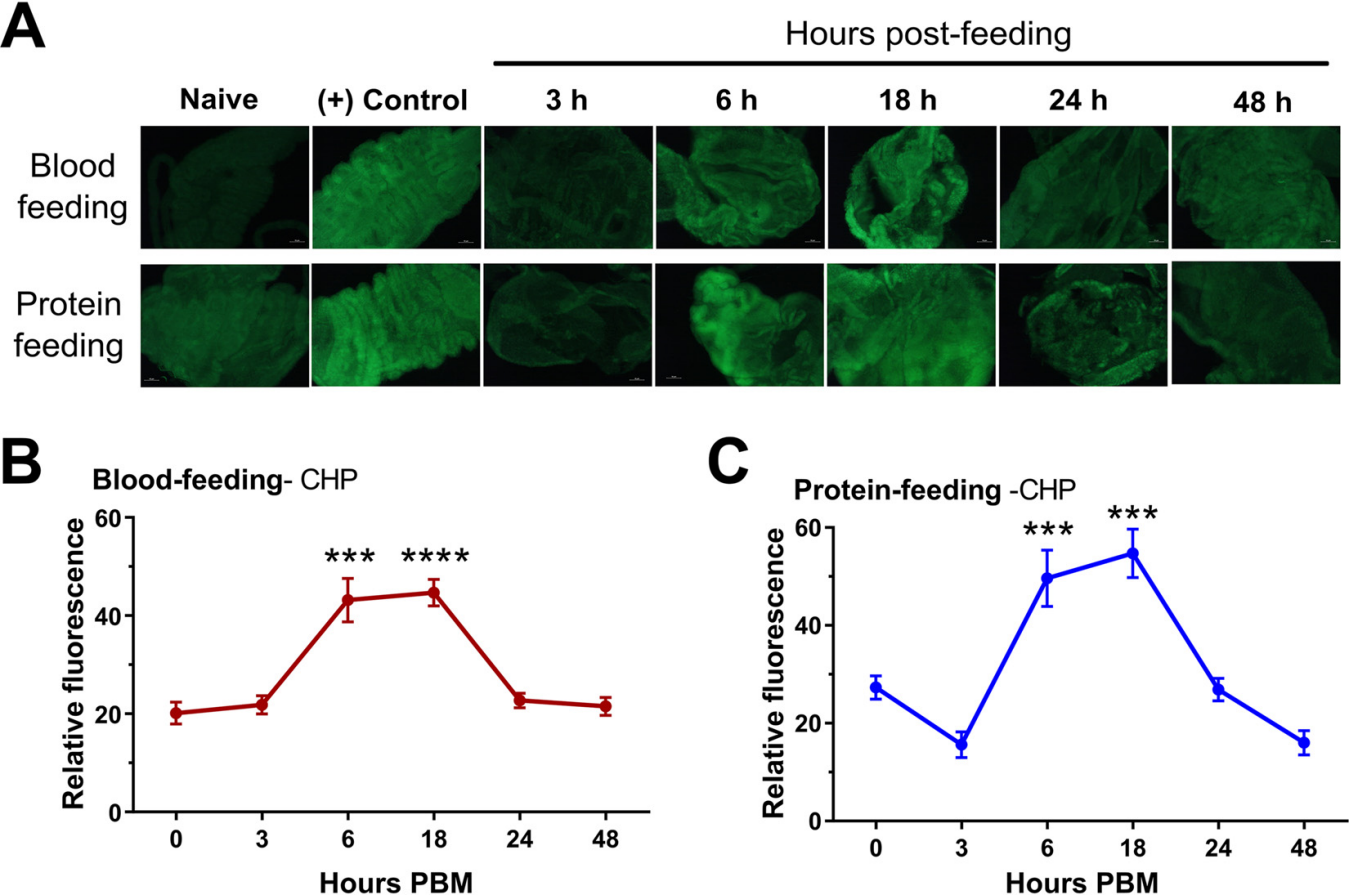
Mosquito feeding promotes the degradation of the midgut basal lamina. Using a fluorescein-labeled collagen hybridizing peptide (CHP) to detect degraded collagen, midgut basal lamina integrity was examined temporally at 3, 6, 18, 24, and 48 h following blood or protein feeding (A). Heat-treated midguts (70°C for 10 min in 1× phosphate-buffered saline [PBS]) were used as a positive (+) control sample. The CHP fluorescence signal was quantified with ImageJ for each sample, and used to determine the relative fluorescence at each time point following blood feeding (B) or protein feeding (C). CHP binding analysis was performed in three independent experiments under blood-fed conditions and in two independent experiments with protein feeding. For each time point, three or more midgut samples were examined by fluorescence microscopy with images analyzed using ImageJ. Relative fluorescence was calculated using the 0-h time point as the baseline measurement, then examined across multiple time points using a one-way analysis of variance (ANOVA) with a Holm-Sidak multiple-comparison test using GraphPad Prism 7 software. Asterisks denote significance (***, *P < *0.001; ****, *P < *0.0001).

*Plasmodium* oocysts develop in the space between the midgut epithelium and the midgut basal lamina (14), providing protection from the cellular or humoral components of the mosquito immune system. While sugar feeding does not distend the mosquito midgut, an additional noninfectious blood meal, as well as the minimal components of a protein meal (BSA and ATP in 1× PBS), promote physical distention of the midgut. To examine if an additional blood or protein meal influences the integrity of the basal lamina, we utilized a collagen hybridizing peptide (CHP) that specifically binds unfolded collagen chains to serve as an indicator of damage to the basal lamina (19, 20). Collagen IV serves as a primary component of the midgut basal lamina (13, 21, 22) that becomes degraded following blood feeding (13). We demonstrate that CHP stains dissected midguts shortly after blood or protein feeding (Fig. 3A), with the intensity of CHP staining reaching peak levels ∼18 h after blood feeding (*P < *0.0001; Fig. 3B) or protein feeding (*P < *0.0001; Fig. 3C) before being quickly repaired thereafter. Together, these results support that the distention of the midgut that accompanies an additional feeding results in the temporary degradation of the basal lamina. Believed to be a protective barrier for developing oocysts (21), this degradation of the basal lamina, albeit temporary, may enable hemolymph immune components or host resources to interact with developing *Plasmodium* oocysts.

### Additional feeding enables TEP1-mediated killing of P. berghei oocysts.

**FIG 4:**
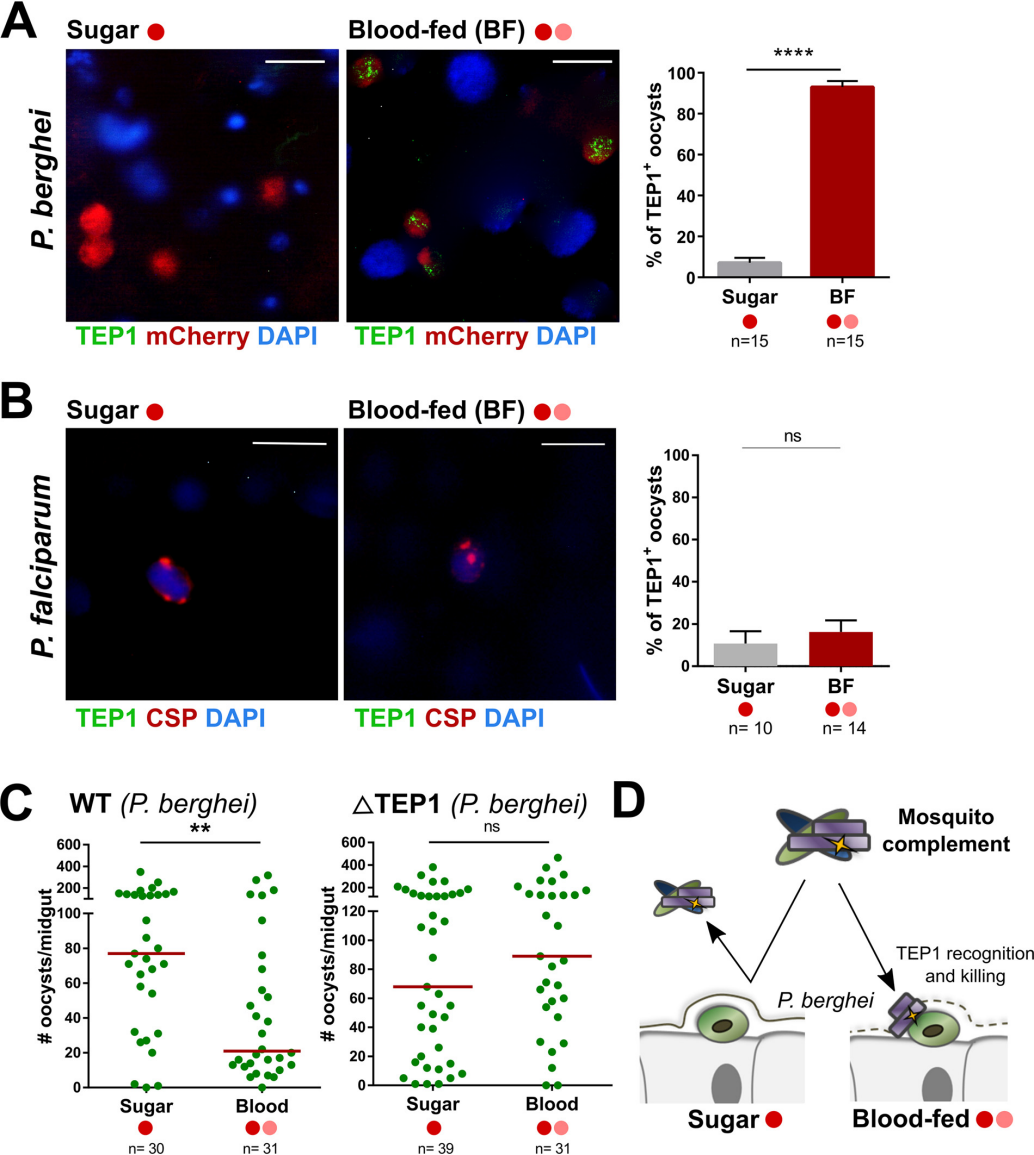
Additional feeding enables the recognition and killing of P. berghei oocysts by mosquito complement. Immunofluorescence assays were performed to examine TEP1 localization on developing oocysts when maintained on sugar or following an additional blood meal. P. berghei oocysts were identified by circumsporozoite protein (CSP) staining and residual signal from the mCherry-parasite background, enabling determination of the percentage of TEP1^+^ oocysts from both experimental conditions (A). Similar experiments were performed following P. falciparum infection (B). Additional feeding experiments were performed on either wild-type (WT) or mutant TEP1 (ΔTEP1) lines to confirm the involvement of mosquito complement in P. berghei oocyst recognition and killing (C). Oocyst numbers were evaluated 8 days postinfection. (D) Model for the role of mosquito complement via TEP1 recognition and killing of P. berghei oocysts. The percentage of TEP1^+^ oocysts for P. berghei and P. falciparum studies are displayed as the mean (+ standard error of the mean [SEM]) and analyzed by Mann-Whitney U test for direct comparison (**, *P < *0.01; ****, *P < *0.0001). Bar, 10 μm. *n*, number of mosquitoes examined; ns, not significant. For each figure, the feeding status is designated by a dark red circle demonstrating an initial P. berghei or P. falciparum parasite infection. Additional blood (pink circle) or protein (blue circle) meals at either 4 days postinfection are designated by their respective colors.

Since an additional feeding (blood or protein meal) during the immature stages of P. berghei oocyst development limits parasite survival (Fig. 1) and degrades the midgut basal lamina independently of the composition of the additional meal (Fig. 3), we hypothesized that the degradation of the basal lamina may expose P. berghei parasites to immune components circulating in the mosquito hemolymph that promote oocyst killing. To address this question, we examined the ability of TEP1, a major determinant of mosquito vector competence that circulates in the hemolymph (23–25), to recognize the newly exposed surface of P. berghei and P. falciparum parasites following an additional blood feeding 4 days postinfection. While not differentially expressed in response to an additional feeding (Fig. 2), using immunofluorescence assays, we demonstrate that TEP1 binds to P. berghei oocysts only after an additional blood meal (*P < *0.0001; Fig. 4A). This suggests that TEP1 is able to access the parasite surface only after the basal lamina is degraded following an additional blood meal. Given the importance of TEP1 and mosquito complement in malaria parasite killing (23–25), TEP1 binding to P. berghei oocysts may account for the significant reduction in P. berghei numbers following an additional feeding at day 4 (Fig. 1). In contrast, blood feeding did not promote TEP1 recognition in similar experiments with P. falciparum (Fig. 4B), suggesting that TEP1 does not recognize P. falciparum oocysts. This is supported by the mechanisms of mosquito complement evasion by P. falciparum ookinetes (26–30) that may extend similarly into the oocyst stage.

To further validate the role of TEP1 and mosquito complement in mediating the P. berghei losses that accompany an additional blood meal, we performed infection and additional feeding experiments (similar to those in Fig. 1) using a TEP1 knockout line of A. gambiae (5, 31). An additional feeding at day 4 promoted P. berghei oocyst killing in a parental wild-type (WT) line, yet these effects were abrogated in the TEP1 mutant, demonstrating an integral involvement for TEP1 in mediating the losses in P. berghei oocyst numbers (*P < *0.01) following an additional blood feeding (Fig. 4C; see also Table S2 in the supplemental materials). However, this is in direct contrast to previous studies demonstrating that TEP1 and mosquito complement are not involved in oocyst killing responses in traditional single-feeding infection experiments (4, 5), therefore arguing that an additional feeding and subsequent degradation of the midgut basal lamina enables TEP1 to recognize and destroy P. berghei oocysts (Fig. 4D).

10.1128/mSphere.00136-21.4 TABLE S2 Raw data for P. berghei infection experiments with wild-type (WT) and TEP1 mutant lines. Download Table S2, XLSX file, 0.01 MB. Copyright © 2021 Kwon et al. 2021 Kwon et al. https://creativecommons.org/licenses/by/4.0/ This content is distributed under the terms of the Creative Commons Attribution 4.0 International license.

### P. falciparum oocysts develop faster when provided with an additional feeding.

**FIG 5:**
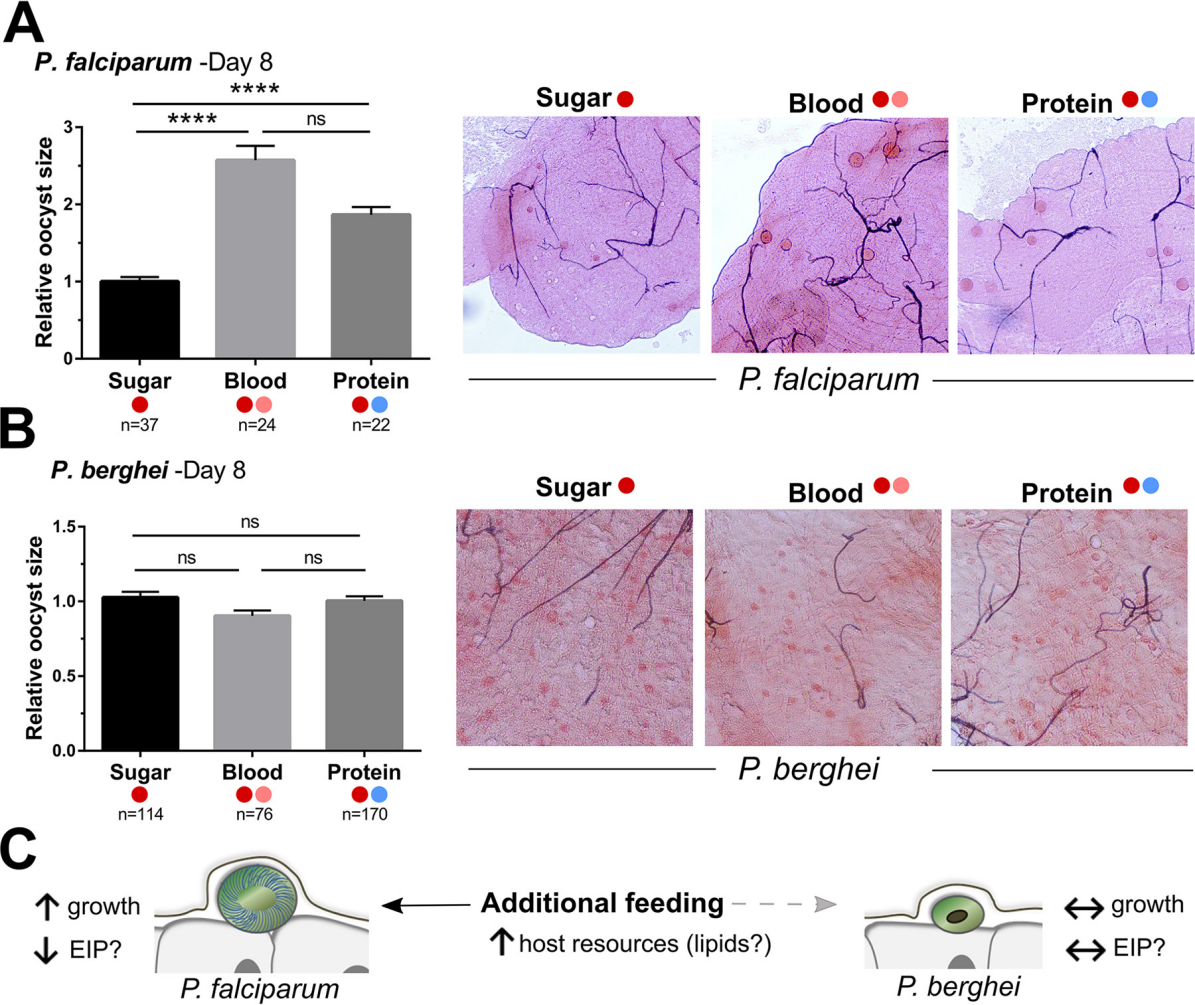
Human malaria parasites utilize host resources provided by an additional feeding to enhance their growth. P. falciparum (A) or P. berghei (B) oocysts were examined at 8 days postinfection. The size of individual oocysts from mosquitoes maintained on sugar or that received an additional blood or protein meal were measured using ImageJ and compared by relative size across conditions. Representative images are shown on the right. Based on growth differences and supporting literature, we propose a model in which human malaria parasites are able to utilize host resources to increase growth and increase the chances of transmission (C). Oocyst measurements were performed in ImageJ using infected midgut micrographs with oocyst measurements in sugar-fed samples from two independent experiments serving as the baseline for relative comparisons using Kruskal-Wallis and Dunn’s multiple-comparison tests in GraphPad Prism 7 software. Asterisks denote significance (****, *P < *0.0001). *n*, number of oocysts examined; ns, not significant. For each figure, the feeding status is designated by a dark red circle demonstrating an initial P. berghei or P. falciparum parasite infection. Additional blood (pink circle) or protein (blue circle) meals at either 4 days postinfection are designated by their respective colors.

Although an additional feeding does not limit human malaria parasite numbers like those of their rodent malaria counterparts (Fig. 1 and 4), when evaluating oocyst numbers, we noticed distinct differences in parasite growth between *Plasmodium* species in the surviving oocysts (Fig. 5). P. falciparum oocysts are significantly larger when mosquitoes receive an additional blood meal (*P < *0.0001) or protein meal (*P < *0.0001) compared to those in mosquitoes maintained on sucrose alone after the infectious blood meal (Fig. 5A). This suggests that human malaria parasites are able to utilize added host resources when provided with an additional feeding to accelerate their growth, as previously suggested (32, 33). Differences in oocyst size between mosquitoes fed a blood or protein meal were not significant (Fig. 5A), suggesting that both substrates are able to initiate comparable changes in mosquito physiology from which parasites can derive host resources (32–35). In contrast to these results with P. falciparum, similar experiments with P. berghei did not influence oocyst size (Fig. 5B), suggesting that rodent malaria parasites are unable to utilize the extra mosquito host resources provided with an additional blood meal at 4 days postinfection (summarized in Fig. 5C). Taken together, our results support a model in which the human malaria parasite has evolved with its natural vector to evade immune recognition (Fig. 4) and to utilize host resources to increase the likelihood of its transmission.

## DISCUSSION

Studies of vectorial capacity in Anopheles spp. have traditionally focused on measurements of *Plasmodium* oocyst or sporozoite numbers to evaluate the potential to transmit malaria. While insightful, these predominantly lab-based studies have not adequately addressed how mosquito physiology influences parasite survival and growth during the approximately 2- to 3-week extrinsic incubation period (EIP). Evidence suggests that larval nutrition (36) and temperature (37, 38) influence the EIP, as well as interactions between different parasite and mosquito species that contribute to vectorial capacity (7, 39). However, our current understanding of the mechanisms that define these host-parasite interactions in the mosquito host are limited. With the importance of blood feeding in shaping mosquito physiology (40–42) and the ability of mosquitoes to feed multiple times during their life span (43, 44), we hypothesized that an additional blood meal may potentially influence the outcomes of an existing malaria parasite infection. As a result, we demonstrate here that an additional blood meal significantly impacts *Plasmodium* development in terms of parasite survival and growth, with stark differences between the abilities of human and rodent malaria parasites to evade immune recognition and to utilize nutrients provided by their mosquito host. These differences in recognition and growth are likely the result of evolution of P. falciparum with A. gambiae as its natural vector, while the laboratory model, P. berghei, has not been under similar selective pressures. However, it is unclear how widely these adaptations extend to other human and rodent malaria systems.

When challenged with an additional blood meal 4 days postinfection (the approximate time to potentially find a new host after completing a gonotrophic cycle), we see a dramatic reduction in the number of P. berghei oocysts, a phenotype recapitulated by similarly feeding on a protein meal. These results suggest that the effects of P. berghei killing are not directly mediated by blood-derived contents but instead rely on either nutritional or physiological signals associated with obtaining a protein-rich meal. Protein serves as an essential building block for mosquito metabolism and the process of vitellogenesis (45), such that an artificial diet containing BSA can promote egg production (16, 17) and expansion of the midgut microbiota comparable to that produced by blood feeding (16, 17). Yet, due to the limitations requiring a protein-based meal to promote midgut distention and the technical challenges of using low-melt agarose (12, 46) with a rodent malaria system, we cannot further delineate the impacts of nutrition on our P. berghei oocyst-killing phenotype.

The ingestion of a blood or protein meal also results in dramatic changes to midgut epithelium cell morphology, causing a flattening of the columnar cells, the loss of microvilli, and substantial degradation of the basal lamina (13, 47). Based on the results of our CHP experiments, we demonstrate that collagen is degraded shortly after taking a blood or protein meal, suggesting that the integrity of the basal lamina is compromised following midgut distention. When paired with immunofluorescence assay (IFA) experiments that illustrate that TEP1 binding to P. berghei oocysts only after an additional feeding, our data support that parasite killing is likely mediated by the degradation of the basal lamina following an additional feeding, enabling the exposure of developing early oocysts to mosquito complement components in the hemolymph. Experiments with a TEP1 mutant line provide further support for the involvement of TEP1 and mosquito complement function in the killing of P. berghei oocysts after an additional blood meal, in contrasting with previous results arguing that TEP1-mediated killing responses only target *Plasmodium* ookinetes (4, 5, 23). This supports a model in which the mosquito basal lamina serves as an integral physical barrier to protect the development of P. berghei from the mosquito innate immune system during oocyst maturation.

Therefore, it is of interest that an additional blood or protein meal does not similarly influence P. falciparum oocysts 4 days postinfection, suggesting that parasites surviving the transition into early oocysts can evade detection by TEP1 and mosquito complement. This is supported by several studies suggesting that in natural parasite-mosquito interactions similar to that of P. falciparum and *A gambiae*, malaria parasites have evolved mechanisms to escape immune recognition (26–30, 48). Previous studies demonstrate that a *Plasmodium* ookinete surface protein, P47, is integral to evading TEP1 and mosquito complement recognition (27–30, 49, 50). Evidence suggests that P47 suppresses JNK signaling that promotes midgut nitration, an essential labeling step required for parasite immune recognition (27, 50). As a result, we speculate that the inability of TEP1 to recognize P. falciparum oocysts is likely due to the continued lack of nitration on parasite surface proteins that mediate complement recognition (50) after these parasites survived ookinete invasion. However, additional evidence suggests that the role of P47 in mosquito complement evasion is incomplete (51) and that other ookinete surface proteins similarly contribute to the evasion of mosquito complement (52). As a result, the mechanisms by which P. falciparum oocysts escape TEP1 recognition remain unknown and warrant further study.

In addition to evaluating the effects of additional blood feeding on parasite survival at 4 days postinfection, we also examined mature oocyst numbers when challenged at 8 days postinfection once parasites have begun sporogony. However, at this later stage of parasite development, an additional blood feeding did not affect either rodent or human malaria parasite numbers. This suggests that there are differences in recognition of early and mature P. berghei oocysts, where only early oocysts are recognized by mosquito complement following an additional blood meal. At present, why mature *Plasmodium* oocysts are no longer susceptible to killing remains unclear. There is support for the hypothesis that turnover of the oocyst capsule during development (14), where both parasite and mosquito proteins present on the oocyst surface are reorganized at the onset of sporogony (53–55), may contribute to these temporal differences. As a result, this turnover of the parasite surface may potentially remove protein(s) involved in mosquito complement recognition of developing immature oocysts.

In agreement with other recent studies (11, 56), we demonstrate that P. falciparum oocysts significantly increase in size following additional feeding. This increased growth suggests that human malaria parasites utilize the added mosquito-derived lipid resources that are provided in an additional blood or protein meal to increase the speed of their development. This can have important implications for malaria transmission, where additional feeding shortens the time of salivary gland infection by sporozoites (11) and increases the number of P. falciparum salivary gland sporozoites (9, 10). This supports the argument that the increased oocyst growth and development that accompanies an additional blood meal may enhance the potential for malaria transmission by significantly reducing the EIP, as previously proposed (11).

This is contrasted by our experiments with P. berghei, where an additional blood or protein meal at 4 days postinfection did not influence oocyst growth. This suggests that P. berghei does not have the same ability as P. falciparum to utilize the additional resources provided with an additional feeding. However, other studies have shown a subtle increase in P. berghei oocyst size when an additional blood meal is provided later in oocyst development at 7 days postinfection (56). As a result, further experiments are needed to resolve whether these observations represent potential differences between *Plasmodium* parasite species or temporal variation in the optimal period for an additional feeding for parasite development.

One caveat of our experimental comparisons between human and rodent malaria parasites are the differences in temperature (25°C versus 19°C) associated with each respective *Plasmodium* system. As a result, we cannot account for the potential impacts of temperature on mosquito host metabolism and physiology that may also contribute to our observed phenotypes for parasite growth.

Together, our experiments argue that human malaria parasites have developed the ability to evade immune detection and to utilize host resources in their natural mosquito vector. This in contrast with our experiments with rodent malaria parasites, which represent a nonnatural system widely used in laboratory studies. Although parasites were reared under different temperatures that undoubtedly influence mosquito physiology, we believe that our data present evidence that *Plasmodium* species have evolved within their mosquito host not only to evade immune detection as previously described (26, 28, 48), but to also exploit resources provided with an additional blood meal to accelerate their development and increase the chances of transmission. As a result, we believe our findings are an important advancement in our understanding of host-parasite interactions and the mechanisms that define vectorial capacity for the transmission of malaria.

Moreover, the influence of an additional blood meal has recently been described in other vector-pathogen systems. In Aedes aegypti and Aedes albopictus, an additional blood meal enhances arbovirus dissemination, increasing the transmission potential of Zika virus (ZIKV), dengue virus (DENV), and Chikungunya virus (CHIKV) (13, 20), while sequential blood feeding in sand flies leads to increased Leishmania parasite numbers and an improved frequency of transmission (57). These examples suggest that blood feeding is a highly conserved, yet relatively unexplored, mechanism for pathogens to take advantage of host blood feeding behaviors to enhance vector-borne disease transmission.

## MATERIALS AND METHODS

### Ethics statement.

The protocols and procedures used in this study were approved by the animal care and use committees at Iowa State University (IACUC-18-228) and Johns Hopkins University (M006H300), with additional oversight from the Johns Hopkins School of Public Health Ethics Committee. Commercial anonymous human blood was used for parasite cultures and mosquito feeding experiments, and therefore human consent was not required.

### Mosquito rearing.

A. gambiae mosquitoes of the Keele strain (58, 59), as well as the TEP1 mutant and parental control X1 lines (5, 31) to examine the role of mosquito complement in oocyst immune recognition, were reared at 27°C with 80% relative humidity and a 14-h/10-h light/dark cycle. At Iowa State University, larvae were fed fish flakes (TetraMin; Tetra), while adult mosquitoes were maintained on 10% sucrose solution and commercial sheep blood for egg production. At the Johns Hopkins Bloomberg School of Public Health, larvae were reared on a diet of fish flakes (TetraMin; Tetra) and cat food (Purina), while adult mosquitoes were fed on anesthetized 6- to 8-week-old female Swiss Webster mice for egg production. The Keele colony at Iowa State was derived from the Keele colony at Johns Hopkins and has been independently maintained for ∼4 years.

### Plasmodium berghei infection.

Female Swiss Webster mice were infected with a P. berghei-mCherry strain as described previously (4, 5). Three- to 5-day-old female mosquitoes were fed on an anesthetized infected mouse displaying active exflagellations. Following challenge, engorged mosquitoes were selected on ice and maintained at 19°C with 80% relative humidity and a 14-h/10-h light/dark cycle. Malaria parasite infection was examined by dissecting individual mosquito midguts in 1× PBS to perform counts of *Plasmodium* oocyst numbers by fluorescence microscopy (Eclipse 50i; Nikon) at either 8 or 10 days postinfection. All P. berghei infections were performed at Iowa State University.

### Plasmodium falciparum infection.

Three- to 4-day-old female mosquitoes were fed through artificial membrane feeders on an NF54 P. falciparum gametocyte culture in human blood as described previously (39). After removal of the unfed females, P. falciparum-infected A. gambiae females were maintained at 27°C on a 10% sucrose solution. Midguts were dissected in 1× PBS and stained in 0.2% mercurochrome to determine oocyst numbers at 8 or 10 days postinfection using a light-contrast microscope. Images were captured using an optical microscope. All P. falciparum infections were performed at the Malaria Research Institute at the Johns Hopkins Bloomberg School of Public Health.

### Additional feeding challenge following *Plasmodium* infection.

Naive female mosquitoes (3 to 6 days old) were initially challenged with a P. berghei-infected mouse or a P. falciparum-infected blood meal. Infected mosquitoes were provided with an egg cup for oviposition, then separated into two groups, one of which was maintained on a 10% sucrose solution for the duration of the experiment. The second group was challenged with either defibrinated sheep blood in the case of P. berghei infection, human blood for P. falciparum infections, or a protein meal (consisting of 200 mg/ml of bovine serum albumin, 2 mM ATP, and 20% [vol/vol] food dye in 1× PBS) for both parasite species using a glass membrane feeder at either 4 or 8 days postinfection. Additional feeding attempts with 0.05%, 0.1%, or 0.2% low-melt agarose in 150 mM NaCl, 20 mM NaHCO3, and 20 mM ATP (12) were unsuccessful, resulting in mosquito mortality within 24 h when kept at 19°C. To assess parasite infection, midgut dissections and oocyst counts were performed at 8 or 10 days postinfection, respectively, to examine the effects of an additional feeding on early or mature oocysts.

Additional experiments performed with the TEP1 mutant and X1 lines were similarly initially infected with P. berghei, then challenged with a naive mouse at day 4 postinfection or maintained on a sucrose diet without receiving a second blood meal. Parasite numbers were evaluated by counting oocyst numbers at day 8 postinfection.

### Measurement of basal lamina integrity using collagen hybridizing peptide.

Following blood or protein feeding, midguts were dissected from mosquitoes after feeding a blood or a protein meal at 3, 6, 18, 24, and 48 h. Midguts from nonfed mosquitoes served as a negative control, while heat-treated midguts (70°C for 10 min) were used as a positive control. After dissection, the blood or protein bolus were removed and the midguts were washed in 1× PBS, then fixed with 2% glutaraldehyde and 2% paraformaldehyde in PBS at pH 7.4 (Electron Microscopy Sciences) for 3 h at 4°C. Following fixation, samples were washed three times in 1× PBS, then blocked overnight at 4°C in blocking buffer (5% bovine serum albumin in 1× PBS). To measure the potential disruption of collagen present on the midgut basal lamina, fluorescein-conjugated collagen hybridizing peptide (CHP) (Echelon Biosciences) that recognizes denatured collagen was diluted in 1× PBS (1:20, 5 μM). Before use, the CHP dilution was placed on a heating block at 80°C for 10 min, then chilled on ice. CHP was added to the blocked midgut samples and incubated overnight at 4°C. Midguts were washed five times in PBS, then mounted with ProLong Diamond Antifade mountant with 4′,6-diamidino-2-phenylindole (DAPI; Life Technologies). Staining was visualized by fluorescence on an Eclipse 50i microscope (Nikon) and captured using NIS Elements (Nikon) imaging software under the same exposure settings. Micrographs were used to quantify fluorescence across samples using ImageJ software (60).

### Relative gene expression.

At 4 days postinfection, P. berghei-infected mosquitoes were challenged with additional blood or protein meals as previously described. To examine the relative abundance of bacteria by measuring levels of bacterial 16S rRNA expression (61), mosquitoes were surface-sterilized at 24 h postfeeding in 75% ethanol for 5 min. Midguts (*n* = 10) were dissected from mosquitoes maintained on sugar and those receiving an additional protein or blood meal. Additional whole mosquito samples (*n* = 5) were collected from each sample condition. Total RNA was extracted from dissected midgut or whole-mosquito samples using TRIzol (Thermo Fisher Scientific) and further purified using the Direct-zol RNA miniprep kit (Zymo Research). Total RNA (200 ng) was used for cDNA synthesis using the RevertAid First Strand cDNA synthesis kit (Thermo Fisher Scientific). qRT-PCR analysis was performed using cDNA (1:5 dilution), 500 nM gene-specific primers, and PowerUp SYBR green master mix (Thermo Fisher Scientific) with the following cycling conditions: 95°C for 10 min, followed by 40 cycles with 95°C for 15 s and 65°C for 60 s. Bacterial 16S rRNA, *Vg*, *TEP1*, and *rpS7* primers are listed in Table S3 in the supplemental material. A comparative threshold cycle (2^−ΔΔ^*^CT^*) method was employed to determine relative transcript abundance for each transcript (62).

10.1128/mSphere.00136-21.5 TABLE S3 Primers used for reverse transcription-quantitative PCR (qRT-PCR) analysis. Download Table S3, DOCX file, 0.01 MB. Copyright © 2021 Kwon et al. 2021 Kwon et al. https://creativecommons.org/licenses/by/4.0/ This content is distributed under the terms of the Creative Commons Attribution 4.0 International license.

### Immunofluorescence assays.

Mosquitoes previously infected with P. berghei were either maintained on 10% sucrose or fed on defibrinated sheep blood at day 4 postinfection. Midguts were dissected ∼24 h after an additional blood feeding or from nonchallenged (sugar-fed) mosquitoes. Midgut sheets were prepared, removing the blood bolus. Midgut samples were washed in 1× PBS before fixation in 4% paraformaldehyde (PFA) for 1 h at room temperature (RT). To examine basal lamina integrity based on staining of the oocyst capsule, midgut samples were washed three times in 1× PBS and then blocked overnight in 1% bovine serum albumin (BSA)/0.1% Triton X-100 in 1× PBS at 4°C. Midgut samples were incubated with a mouse monoclonal 2A10 anti-Plasmodium falciparum circumsporozoite protein (CSP) antibody (1:500; BEI Resources) and rabbit-TEP1 (1:500) primary antibodies overnight in blocking buffer (1% BSA/1× PBS) at 4°C. After washing in 1× PBS, midguts were incubated with Alexa Fluor 488 goat anti-rabbit IgG (1:500; Thermo Fisher Scientific) and Alexa Fluor 568 goat anti-mouse IgG (1:500; Thermo Fisher Scientific) secondary antibodies in blocking buffer for 2 h at RT. Due to the use of transgenic mCherry parasites, the CSP signal could not be specifically distinguished. Midguts were washed three times in 1× PBS, then mounted with ProLong Diamond Antifade mountant with DAPI for visualization. To quantify TEP1-positive P. berghei oocysts, 20 oocysts were randomly selected from individual mosquito midguts, and the percentage displaying TEP1^+^ positive oocysts was recorded. Data were compiled from two independent experiments.

For P. falciparum infection experiments, mosquitoes were maintained on 10% sucrose or fed on noninfected human blood 4 days postinfection. Midguts were dissected ∼24 h after an additional blood feeding or from nonchallenged (sugar-fed) mosquitoes. The blood bolus was removed, and the midgut samples were washed in 1× PBS before fixation in 4% PFA for 24 h during overnight shipping. Midgut sheets were prepared for both and then washed three times in 1× PBS. Samples were then blocked overnight in 1% bovine serum albumin (BSA)/0.1% Triton X-100 in 1× PBS at 4°C, then incubated overnight as above with a mouse monoclonal 2A10 anti-P. falciparum CSP antibody (CSP, 1:500) and rabbit-TEP1 (1:500) primary antibodies in blocking buffer (1% BSA/1× PBS) at 4°C. After washing in 1× PBS, midguts were incubated with Alexa Fluor 488 goat anti-rabbit IgG (1:500; Thermo Fisher Scientific) and Alexa Fluor 568 goat anti-mouse IgG (1:500; Thermo Fisher Scientific) secondary antibodies in blocking buffer for 2 h at RT. Midguts were washed three times in 1× PBS, then mounted with ProLong Diamond Antifade mountant with DAPI for visualization. To quantify TEP1-positive P. falciparum oocysts, all stained oocysts were examined on individual mosquito midguts, and the percentage of TEP1^+^ positive oocysts was recorded.
