# Supplementary figures and images for: Additional Feeding Reveals Differences in Immune Recognition and Growth of Plasmodium Parasites in the Mosquito Host
Source: mSphere. 2021 Mar 31;6(2):e00136-21. doi: 10.1128/mSphere.00136-21 (PMC8546690; doi:10.1128/mSphere.00136-21)

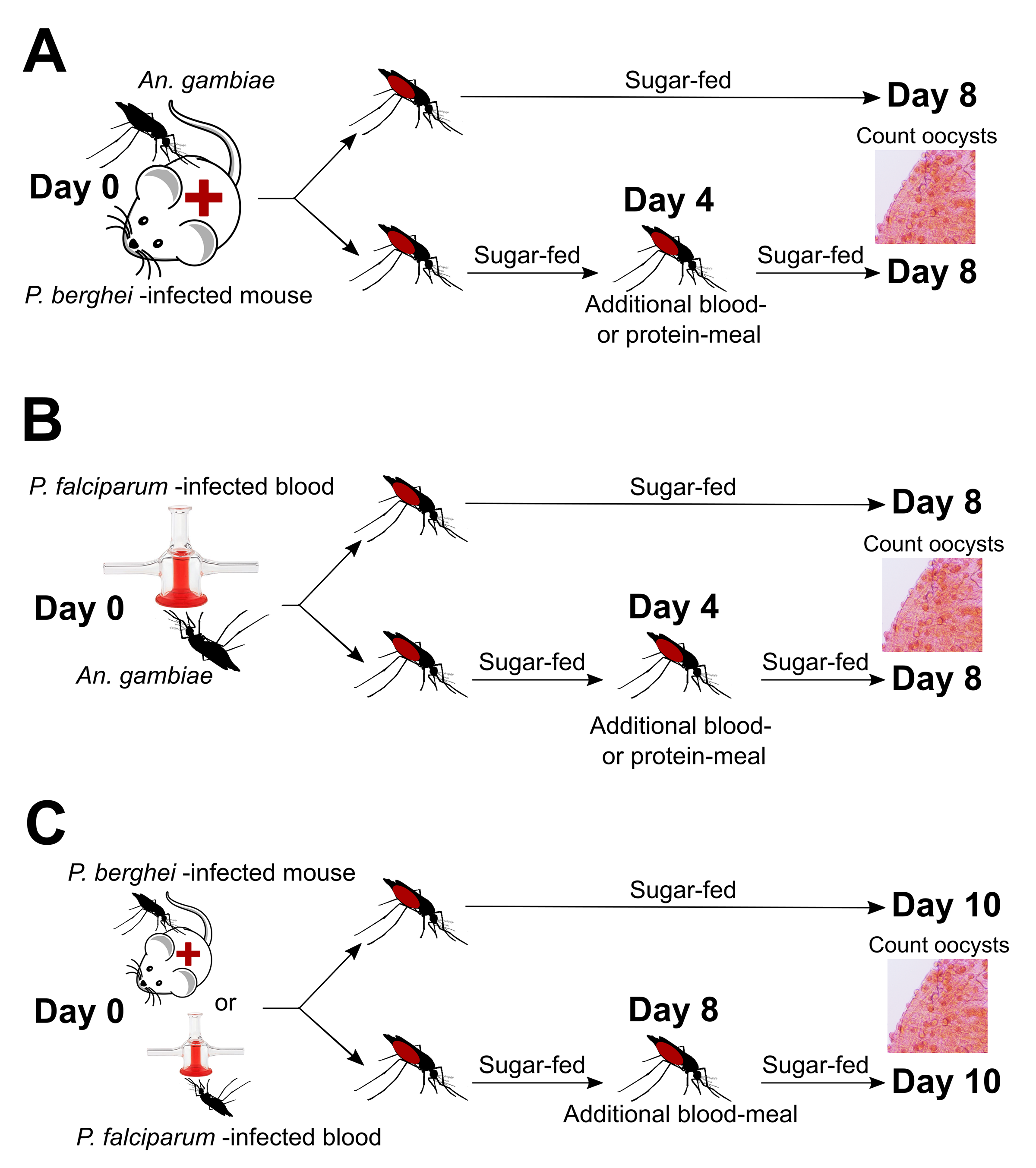

Supplement: FIG S1 [file msphere.00136-21-sf001.tif]
