# Supplementary material for: Additional Feeding Reveals Differences in Immune Recognition and Growth of Plasmodium Parasites in the Mosquito Host
Source: mSphere. 2021 Mar 31;6(2):e00136-21. doi: 10.1128/mSphere.00136-21 (PMC8546690; doi:10.1128/mSphere.00136-21)
Supplement: TABLE S3 [file msphere.00136-21-st003.docx]

**Table S3. Primers for qRT-PCR analysis**

**Primer Gene ID Sequence (5’- 3’)**

rpS7-F AGAP010592 ACCCCATCGAACACAAAGTTGACACT

rpS7-R CTCCGATCTTTCACATTCCAGTAGCAC

Universal bacteria 16S-F N/A TCCTACGGGAGGCAGCAGT

Universal bacteria 16S-R GGACTACCAGGGTATCTAATCCTGTT

Vg-F AGAP004203 TGCAGTACATCGAGCAGGGTGACAA

Vg-R CTTGACGGTCTTGGTGACCGACTTG

TEP1-F AGAP010815 CAGACAGATGGTTCGTTTGGTGTG

TEP1-R CCAGCAATGCCGTCAACACATAC
